# Venocentric perspective on varicocele: summarizing mechanisms and explorations

**DOI:** 10.3389/fphys.2026.1828242

**Published:** 2026-05-29

**Authors:** Qianbei Chen, Xingbo Wang, Guicheng Liu, Xiaojin Zhang, Zhuomin Xu, Weiqing Xu, Xujun Yu, Liang Dong

**Affiliations:** 1 School of Medical and Life Sciences, Chengdu University of Traditional Chinese Medicine, Chengdu, China; 2 Traditional Chinese Medicine (TCM) Regulating Metabolic Diseases Key Laboratory of Sichuan Province, Hospital of Chengdu University of Traditional Chinese Medicine, Chengdu, China; 3 Chengdu University of Traditional Chinese Medicine, Chengdu, China; 4 The Second Affiliated Hospital of Chengdu University of Traditional Chinese Medicine, Chengdu, China

**Keywords:** endothelial dysfunction, oxidative stress, varicocele, vascular remodeling, venous hemodynamics

## Abstract

Varicocele (VC), one of the most common correctable etiologies of male infertility, has long been pathophysiologically characterized by hemodynamic disturbances stemming from venous valvular incompetence and the consequent testicular insult. Yet this macrovascular perspective demonstrates limited explanatory power in accounting for the pronounced clinical heterogeneity of VC, the inter-individual variability in testicular impairment, and the nonlinear recovery of fertility following microsurgical repair. This review endeavors to present a propositional shift in perspective—one that warrants further empirical validation—drawn from vascular biology: between the inciting hemodynamic disturbance and the resultant testicular injury, an adaptive remodeling imbalance of the venous wall may occupy a position of considerable pathophysiological importance. Within this conceptual framework, the role of the spermatic vein might be extended from that of a passive conduit subjected to pressure toward that of a functional organ actively engaged in local microenvironmental regulation. This process involves a cascade of events, including endothelial dysfunction, phenotypic transition of vascular smooth muscle cells, progressive dysregulation of the collagen-to-elastin ratio within the extracellular matrix, and aberrant activation of pro-inflammatory and pro-fibrotic signaling within the vessel wall. Should this hypothesis prove tenable, such a paradigm transition would facilitate a reappraisal of VC-associated testicular damage through the microscopic lens of venous wall exhaustion, and offer a potential theoretical supplement and integrative framework for exploring disease-staging strategies and interventional timing that transcend conventional palpation-based grading, predicated instead upon vascular biological features of the venous wall.

## 1 Introduction

Varicocele (VC) is characterized by abnormal dilation and tortuosity of the pampiniform plexus and represents a significant cause of male infertility (Jensen et al., 2017). It affects approximately 15% of adult males, 35% of men with primary infertility, and up to 80% of those with secondary infertility (Alsaikhan et al., 2016). The spermatic vein is formed by the anterior, middle, and posterior interconnected venous networks from the testis, epididymis, and spermatic cord, collectively constituting the pampiniform plexus, which ultimately drains into the external inguinal ring and two venous branches. The primary functions of the spermatic vein encompass venous drainage of the testis, thermoregulation via the pampiniform plexus, and hormone transport. Dysfunction in these roles constitutes the pathophysiological foundation of VC formation and its associated infertility (Zampieri, 2021; De Toni et al., 2022).

**Figure 1:**
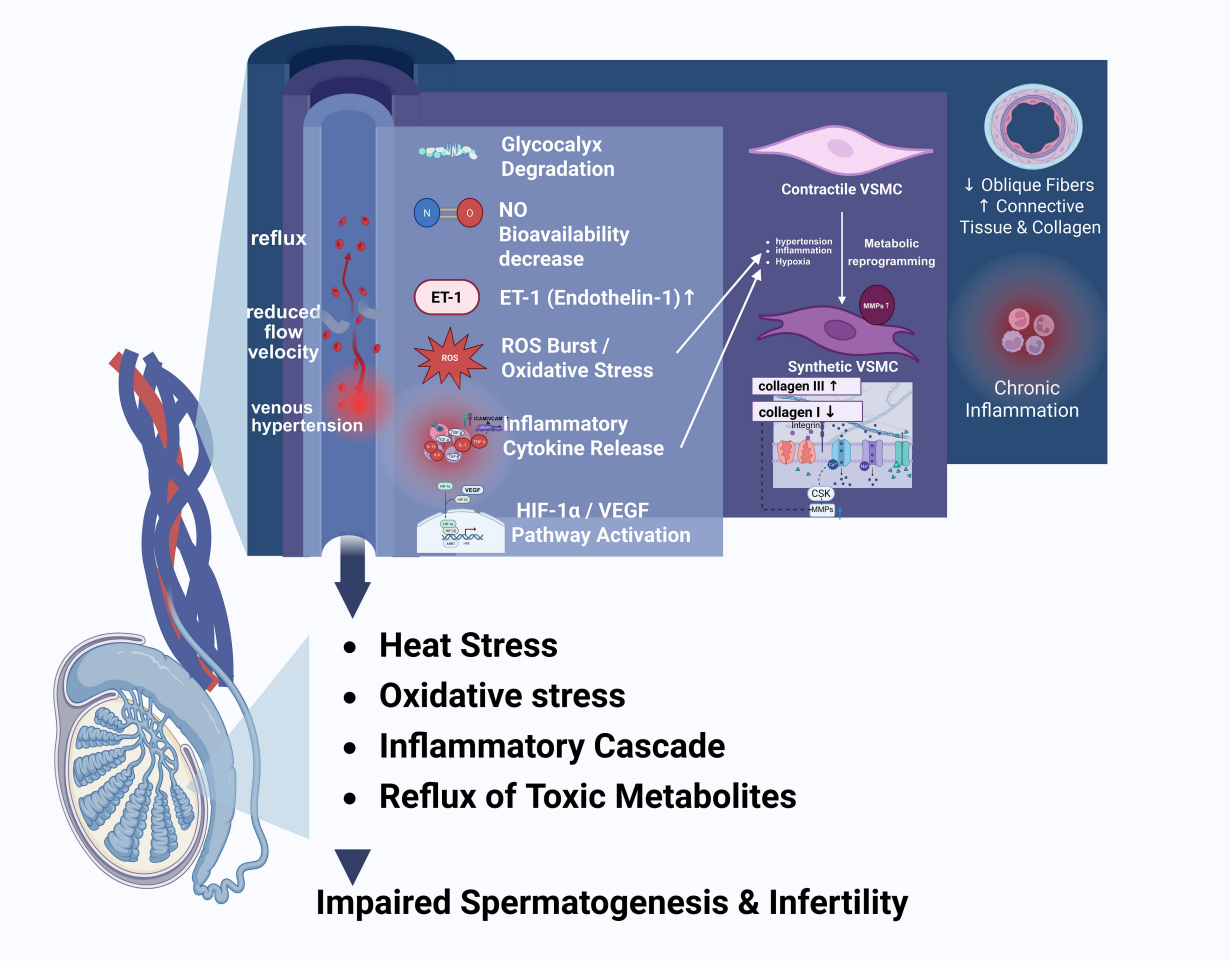
Venocentric pathogenesis of varicocele: a vicious cycle of hemodynamic stress and tri-laminar venous wall remodeling. Hemodynamic stress (reflux/hypertension) initiates tri-laminar venous wall remodeling. Intimal endothelial dysfunction (ROS/inflammation), medial VSMC phenotypic switching (ECM dysregulation), and adventitial fibrosis establish a vicious cycle that exacerbates valvular incompetence. Ultimately, venous wall failure leads to impaired testicular microcirculation, oxidative stress, and male infertility.

The traditional theory attributes VC to the right−angle insertion of the left spermatic vein into the left renal vein, combined with valvular incompetence, leading to reflux, venous hypertension, and ultimately testicular injury. However, this model cannot explain why individuals with similar anatomy and hemodynamics differ markedly in disease progression or why some patients respond poorly to surgery (Shabana et al., 2015; Samplaski and Jarvi, 2016). These discrepancies suggest that, beyond macroscopic flow disturbances, microscopic properties of the venous wall itself play a decisive role. The vein is not a passive conduit but an active three−layered structure (intima, media, adventitia) that undergoes adaptive or pathological remodeling in response to hemodynamic stress (Studennikova et al., 2019). As seen in **Figure 1**, we propose an integrated paradigm: VC results from a vicious cycle between macroscopic hemodynamic disturbances and defective layer−specific responses of the venous wall. Hemodynamic stress triggers the process, but progressive dysfunction and remodeling of the intima, media, and adventitia drive disease progression and testicular damage.

## 2 Hemodynamic derangements

Venous valvular incompetence and reflux cause hemodynamic derangements in VC, characterized by blood flow obstruction and subsequent venous hypertension. Zou, Bagheri (Bagheri et al., 2018), and colleagues have delineated ultrasonographically the hemodynamic derangements triggered by valvular incompetence (Zou et al., 2025). This pathological cascade begins with transient reflux during the Valsalva maneuver (Grade 1) and, with progressive reflux and compensatory collateral dilation, progresses to persistent reflux at rest (Grade 3) and ultimately results in complete hemodynamic stasis (Grade 4). These observations highlight how primary venous valvular insufficiency directly causes abnormalities in blood flow velocity, direction, and pressure. The resulting aberrant shear stress and sustained venous hypertension constitute the initial physical signals for venous wall pathology. These forces directly drive venous wall remodeling—including dysregulated extracellular matrix (ECM) metabolism, smooth muscle cell dysfunction, and endothelial injury. Concurrently, the high-pressure, hypoxic environment caused by blood stasis serves as a critical trigger of oxidative stress and inflammatory responses in endothelial cells and other cellular components.

Furthermore, in the context of composite etiologies such as the nutcracker phenomenon (Kurklinsky and Rooke, 2010), valvular incompetence acts as a pivotal conduit, transmitting upstream hypertension to the spermatic venous system. Without failure of this critical juncture, renal vein hypertension seldom induces clinical VC. Studies confirm that hypertension resulting from left renal vein compression must retrograde through an incompetent left spermatic vein valve to reach the pampiniform plexus and induce varicosity (Masson and Brannigan, 2014; Lewis et al., 2015). Thus, valvular function serves as the key intrinsic determinant for whether renal venous hypertension progresses to overt VC. Hemodynamically induced alterations in shear stress are closely linked to weakening of the venous wall and its valves, and the study of venous wall remodeling is of considerable relevance to conditions such as Nutcracker Syndrome.

Hemodynamic assessment provides value beyond diagnosis; it identifies the origin and magnitude of reflux, thereby delineating the pathophysiological stage. The hemodynamic disturbances initiated by valvular incompetence establish an aberrant mechanical microenvironment, which, in turn, activates and integrates downstream mechanisms—including venous wall remodeling, oxidative stress, and inflammatory responses—culminating in testicular functional impairment.

## 3 Structure and function of normal blood vessels

Veins, as capacitance vessels, exhibit a thin-walled structure comprising three distinct layers: the tunica intima (endothelial cells), tunica media (smooth muscle cells), and tunica adventitia (fibroblasts). These cellular components are interconnected and structurally supported by an ECM rich in collagen and elastin fibers (Yetkin and Ozturk, 2018).

The three venous wall layers may synergize under mechanical stress to maintain homeostasis. The endothelium forms a selective barrier via tight junctions and secretes nitric oxide (NO) and PGI2 for antithrombotic, vasomotor, and anti-inflammatory effects (Poredos et al., 2021). The media includes smooth muscle cells that actively regulate diameter, elastic fibers that provide passive recoil, and collagen fibers that limit overdistension to ensure integrity. The adventitia (fibroblasts, vasa vasorum, nerves) provides support, nutrition, and neural regulation, and participates in chronic remodeling (Ortega et al., 2021).

It is widely believed that, in response to shear stress or intraluminal pressure changes, the intima senses signals and releases vasoactive substances; the media adjusts contraction and recoil; and the adventitia provides external support and may undergo fibrosis under prolonged stimulation (Pfisterer et al., 2014). Any layer abnormality disrupts this balance, triggering a vicious cycle of endothelial dysfunction, oxidative stress, and inflammation, ultimately leading to venous valvular insufficiency and dilation and, in turn, chronic venous disease (CVD) (Pfisterer et al., 2014; Castro-Ferreira et al., 2018). This may be similar in VC. Yetkin and Ileri proposed that lower extremity varicose veins, hemorrhoids, VC, and pelvic venous congestion may share similar pathophysiological steps (Yetkin and Ileri, 2016). Clinical data from Qiu et al. support this notion: the incidence of VC is significantly higher in patients with lower extremity varicose veins (x²=20.05, p<0.01), and the maximum diameter of the spermatic vein positively correlates with that of lower extremity varicose veins (r=0.4072, p< 0.01) (Qiu et al., 2017). Oztekin et al. found that brachial artery flow-mediated dilation (FMD) was significantly lower in the VC group than in controls among 128 subjects, and multivariate regression analysis identified FMD as an independent correlate of VC (Oztekin et al., 2019). This indicates that patients with VC exhibit not only local venous wall pathology but also systemic endothelial dysfunction. Although we have linked and extrapolated CVD to VC, we recognize that there are inevitable differences in their pathophysiological details. Accordingly, we will review the evidence about the VC layer by layer below.

## 4 Intimal pathology: the initiating step of functional inactivation

**Figure 2:**
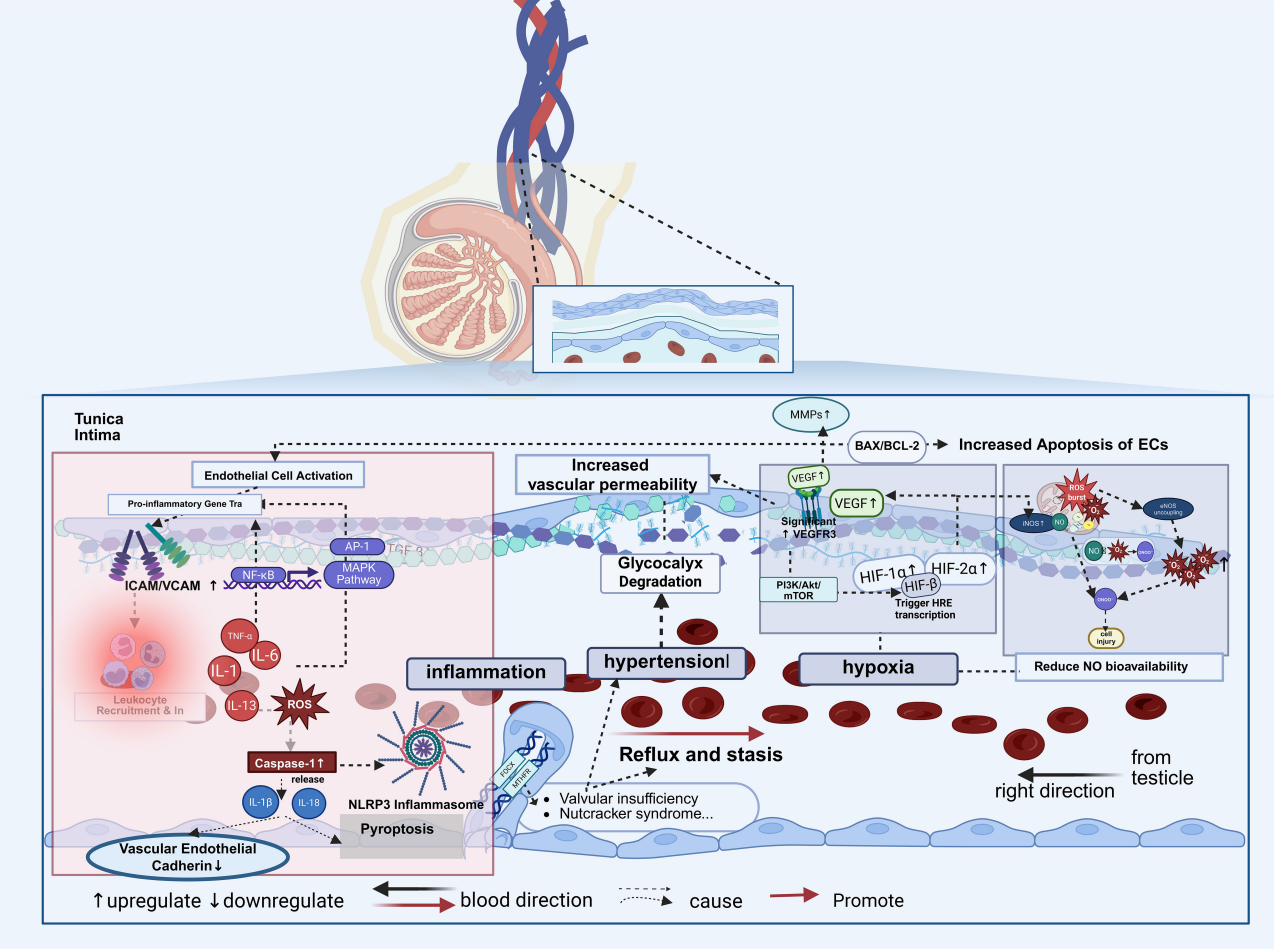
main potential mechanisms of endothelial dysfunction in varicocele. Endothelial dysfunction in varicocele arises from synergistic pathological interactions under hemodynamic stress, hypoxia, and inflammation. Hemodynamic abnormalities (reflux, hypertension) and chronic hypoxia trigger oxidative stress via mitochondrial dysfunction and NOX activation, generating excess ROS. Concurrently, eNOS uncoupling due to BH4 depletion shifts NO production to superoxide, while ROS rapidly scavenge NO to form cytotoxic peroxynitrite. Glycocalyx degradation by MMPs/heparanase further increases permeability and leukocyte adhesion. Inflammatory activation upregulates adhesion molecules (VCAM-1/ICAM-1) and cytokines (TNF-α/IL-6), perpetuating endothelial injury. These processes collectively reduce NO bioavailability, impair vasoreactivity, and compromise microcirculation, ultimately contributing to testicular dysfunction.

Low and disturbed shear stress, primarily sensed by vascular endothelium, is transduced into biochemical signals via various mechanosensors. **Figure 2** zooms in on the specific molecular cascades operating at the endothelial interface. This process, directly or indirectly, induces oxidative stress and triggers inflammatory responses, collectively causing endothelial dysfunction characterized by impaired selective permeability, dysregulated vasomotor tone, and diminished anticoagulant, anti−inflammatory, band anti−proliferative capacities (Boulanger, 2016).

### 4.1 Impaired barrier function and increased permeability

The glycocalyx is a carbohydrate-rich layer on the endothelial surface, consisting of glycoproteins, proteoglycans, and glycosaminoglycans (GAGs) (Reitsma et al., 2007). It acts as both a selective molecular sieve and a mechanosensor, converting shear stress into biochemical signals that regulate NO release, vascular tone, and inflammation (Reitsma et al., 2007). Its integrity is essential for inhibiting leukocyte adhesion and maintaining endothelial barrier function (Pries et al., 2000).

Growing evidence indicates that glycocalyx disruption is a key mechanism in CVD (Ligi et al., 2018). Venous hypertension and disturbed flow due to valvular incompetence activate matrix metalloproteinases (MMPs) and heparanase, degrading glycocalyx components (Zieliński et al., 2024). This loss enhances endothelial permeability, promotes leukocyte adhesion, upregulates inflammatory mediators and adhesion molecules, and induces smooth muscle cell phenotypic switching and ECM remodeling—collectively driving venous dilation and progression to varicose veins (Mannello et al., 2013). Notably, glycocalyx restoration has been proposed as a therapeutic target for CVD. Sulodexide, a glycosaminoglycan blend, has demonstrated glycocalyx-repairing, MMP-9-inhibiting, venotonic, anti-inflammatory, and ulcer-healing effects, supporting its potential in CVD management. However, no studies have specifically examined the state of glycocalyx molecules in spermatic veins, which may play a significant role in the early restoration of venous function.

### 4.2 Changes in vasoactive substance expression

In general endothelial physiology, NO is generated from L-arginine by endothelial nitric oxide synthase (eNOS), whose activity is regulated by shear stress, acetylcholine, bradykinin, and mechanical strain. NO diffuses into vascular smooth muscle cells (VSMCs), activates soluble guanylyl cyclase (sGC), which catalyzes the conversion of GTP to cGMP. cGMP activates cGMP-dependent protein kinase (PKG) to phosphorylate target proteins, leading to reduced intracellular calcium levels, smooth muscle relaxation, vasodilation, and decreased vascular tone (Wang and He, 2024).

Multiple studies agree that NO levels are elevated in dilated veins, whereas the release of endothelium-derived NO may be reduced (Mitropoulos et al., 1996; Romeo et al., 2001; Yildiz et al., 2003). This paradox may be explained by reduced physiological NO production due to endothelial dysfunction, alongside pathological NO release mediated by inducible nitric oxide synthase (iNOS) overexpression in inflammatory cells and compensatory NO secretion from other sources. Consistently, studies in experimental rat models of VC have demonstrated elevated NO levels that directly correlate with upregulated iNOS expression (De Stefani et al., 2005). iNOS overexpression is likely mediated by hypoxia-inducible factor (HIF), as iNOS has been identified as a direct downstream target of HIF-1α (Li et al., 2022). Under physiological conditions, iNOS expression is minimal in endothelial cells (Kone et al., 2003). However, under pathological stimuli such as HIF-1α activation, iNOS is markedly upregulated, accelerating the depletion of L-arginine and tetrahydrobiopterin (BH4), thereby further compromising NO bioavailability (Cinelli et al., 2020). In other words, the initial oxidative stress may trigger a vicious cycle: BH4 depletion induces NOS uncoupling, and the uncoupled NOS in turn becomes a significant new source of reactive oxygen species (ROS), further amplifying oxidative stress while directly impairing functional NO production. Studies have also shown that eNOS gene polymorphisms may contribute to VC pathogenesis by reducing NO levels. Conversely, elevated NOS3 expression in pathological states may represent a compensatory dilatory mechanism to preserve vascular integrity in VC (Kahraman et al., 2016). Although preliminary studies suggest a role for iNOS activity in VC-associated testicular dysfunction, whether NO in the spermatic vein primarily originates from iNOS upregulation induced by oxidative stress or inflammation and how this relates to endothelial dysfunction requires further investigation (Santoro et al., 2001; Kisa et al., 2004; Türker Köksal et al., 2004). Nevertheless, it should be noted that H_2_S exhibits a complex interplay with NO by protecting eNOS from oxidative inactivation and uncoupling, scavenging ROS, and modulating the cGMP pathway to promote vasorelaxation (Smimmo et al., 2024). This implies that the administration of antioxidants serves not merely to scavenge ROS, but crucially to disrupt the vicious cycle of eNOS uncoupling and to preserve NO bioactivity (Barati et al., 2020).

Additionally, asymmetric dimethylarginine (ADMA), an endogenous eNOS inhibitor, competitively inhibits eNOS and reduces NO synthesis when elevated. In VC patients, ADMA levels may be increased in both plasma and seminal plasma, further compromising NO bioavailability (Kiziler et al., 2015).

The study by Yildiz et al. also demonstrated a stepwise deterioration in the response of diseased veins to vasoactive substances across different VC grades. Grade III VC veins exhibited significantly lower maximal contractile responses to phenylephrine, 5-HT, and histamine compared to lower-grade groups (P < 0.05), indicating increased vasoconstrictor reactivity. cGMP content in grade II/III VC tissues decreased by more than 65% relative to grade I, suggesting severe impairment of the NO−cGMP signaling pathway. The potentiating effect of L−NAME on phenylephrine−induced contraction was markedly attenuated in grade III VC, confirming progressive endothelial dysfunction. Although the ex vivo design of this study precluded simulation of shear stress, potentially underestimating endothelial function, and the use of cGMP as a surrogate for NO may be confounded by factors such as sGC activity, it nevertheless underscores the need to examine grade-dependent changes to better understand the progressive nature of the disease. In adolescent VC patients, elevated NO levels are strictly localized to the spermatic venous system; whereas in adult patients, this nitrosative stress has extended to the testicular parenchyma (Mason and Clavijo, 2023). This observation also reveals the progressive nature of VC pathophysiology. It suggests that, beyond the imbalance between NO and ADMA, other vasoactive factors—such as endothelin and prostacyclin—may also be altered, affecting endothelial and vascular function. Future investigations should focus on dynamic changes in local endothelial injury and dysregulation of vasoactive mediators in the spermatic vein in relation to VC grade. However, studies in this area remain lacking.

Immunohistochemical analysis reveals significant upregulation of ET-1, ETA, and ETB receptors within VC spermatic veins (Gyftopoulos et al., 2011). This contrasts with the downregulation reported in lower extremity varicosities (Okuyama et al., 1988; Agu et al., 2002). Hemodynamic shear stress and elevated intraluminal pressure are proposed triggers for this compensatory ET-1/ET receptor axis activation (Gyftopoulos et al., 2011). ET-1, acting predominantly via ETA receptors, stimulates VSMC proliferation, contributing to medial/intimal hyperplasia and the characteristic structural derangements of varicose veins (Gyftopoulos et al., 2011). Local hypoxia from venous stasis may also provoke this secondary endothelin activation (Gyftopoulos et al., 2011). Circulating endothelin levels are reported to be elevated in VC patients (Agu et al., 2002; Gyftopoulos et al., 2011).

### 4.3 A platform for the initiation of oxidative stress and inflammation

Blood stasis leads to local tissue hypoxia, which further disrupts normal endothelial function. Excess ROS generated under high oxidative stress attacks sperm DNA, membranes, and proteins, impairing sperm function and inducing apoptosis, ferroptosis, pyroptosis, and necrosis. Consistent evidence shows elevated ROS levels in infertile patients with VC, indicating a pathological role of ROS in spermatogenic impairment (Yoon et al., 2010). Earlier studies found that, compared with peripheral veins, the spermatic vein exhibits higher activities of NO synthase and xanthine oxidase, as well as elevated levels of NO and peroxynitrite. Serum antioxidant capacity was stronger in the dilated spermatic vein than in peripheral veins, whereas erythrocyte antioxidant capacity was weaker, consistent with an increased rate of peroxynitrite formation. These findings suggest that the release of NO synthase and xanthine oxidase in the dilated spermatic vein contributes to elevated oxidative stress (Mitropoulos et al., 1996). Other studies have also indicated elevated levels of oxidative stress markers (e.g., malondialdehyde, MDA) alongside reduced antioxidant enzyme activity (Ozbek et al., 2008; Blumer et al., 2012). Although ROS-induced testicular damage is well established, direct evidence, such as immunohistochemistry for ROS in the spermatic vein endothelium, remains lacking.

Under hypoxic stress, damaged endothelial cells also overexpress various factors, such as vascular endothelial growth factor (VEGF), to compensate by promoting angiogenesis. This directly demonstrates that the intima actively responds to and attempts to remodel the pathological environment. A study of infertile men with VC directly examined the expression of VEGF and its receptors (VEGFR1, VEGFR2, VEGFR3) and found that they are abnormally activated in these patients (Nazari et al., 2020). Another protein expression analysis comparing varicocele veins with healthy control vessels further confirmed this conclusion. Also, it revealed an imbalance in the ratio of the pro-apoptotic protein BAX to the anti-apoptotic protein BCL-2, indicating that activation of the VEGF signaling pathway is closely associated with dysregulated endothelial cell apoptosis (Abedinzadeh et al., 2025).

In the testis, hypoxia upregulates HIF-1α, which activates downstream VEGF signaling. VEGF then mediates PI3K/Akt signaling, leading to spermatogenic cell apoptosis, disruption of seminiferous tubule structure, and impaired spermatogenesis. Silencing HIF-1α inhibits the VEGF/PI3K/Akt pathway, reverses these pathological changes, and improves spermatogenic function in VC rats (Wang et al., 2021). Studies have confirmed that HIF-1α expression is significantly increased in the spermatic vein. We speculate that VEGF may damage the vascular endothelium through a similar mechanism, although whether additional signaling pathways are involved requires further targeted investigation (Lee et al., 2006).

One study found that serum VEGF levels in adolescents with VC did not change significantly during the observation period, which appears to contradict many other reports (Pichugova et al., 2023). However, this discrepancy may be attributed to the instability of serum measurements and the possibility that pathophysiological changes in adolescents have not yet progressed to a stage causing a marked systemic increase in VEGF. Overall, the theory of elevated VEGF remains credible, and measuring VEGF or VEGFR levels in the endothelium of adolescents with different grades of VC may resolve this contradiction.

More critically, activated endothelial cells release a variety of pro-inflammatory cytokines. In the experimental left-sided VC-induced testicular injury model, IL-1β expression was significantly increased (Papadimas et al., 2002). Targeted studies have found that IL-6 levels in blood samples drawn from the sperm cords of the VC were considerably higher than those in the homozygous peripheral blood and healthy control groups, thereby directly demonstrating the presence of local inflammation (Lattimer et al., 2016). Similarly, TNF-α levels in patients with VC also significantly increased (Micheli et al., 2019). As noted, physical stimuli activate vascular endothelial cells, leading to upregulated adhesion molecules and recruitment of inflammatory cells, including neutrophils and macrophages. These infiltrating immune cells, along with activated vascular wall cells, release pro-inflammatory cytokines such as TNF-α. TNF-α mediates its effects primarily through activation of the canonical NF-κB and MAPK (e.g., p38) pathways, with phosphorylation and nuclear translocation of NF-κB p65 representing key activation events. It can initiate the transcription of a series of inflammation-related genes, including the expression of adhesion molecules such as ICAM-1 and VCAM-1 (Zhang et al., 2021), further amplifying the inflammatory response. TNF-α and IL-6 are also likely to amplify oxidative stress and reduce NO utilization by inducing endothelial cells to produce ROS through the mechanism described above, which has been validated in cerebrovascular and oral epithelial cells (Sprague and Khalil, 2009; Kalliolias and Ivashkiv, 2016). Studies have shown that high levels of cell damage induced by ROS can lead to inflammation in VC rats (Razi et al., 2012). ROS can also promote the expression of pro-inflammatory cytokines and chemokines by activating the classical and non-classical pathways of NF-κB, disrupting feedback regulation to maintain inflammation (Rochfort et al., 2014).

Sustained inflammation and oxidative stress transmit downstream signals, potentially leading to vascular wall remodeling and progressive weakening of the venous wall. For instance, elevated VEGF binds to its receptor (particularly VEGFR2) and activates a series of pro-angiogenic programs, including endothelial cell proliferation, migration, lumen formation, and the secretion of MMPs to degrade the basement membrane and promote the invasion of new vessel sprouts (Nazari et al., 2020).

## 5 Media pathology: core of active tension loss and passive remodeling

**Figure 3:**
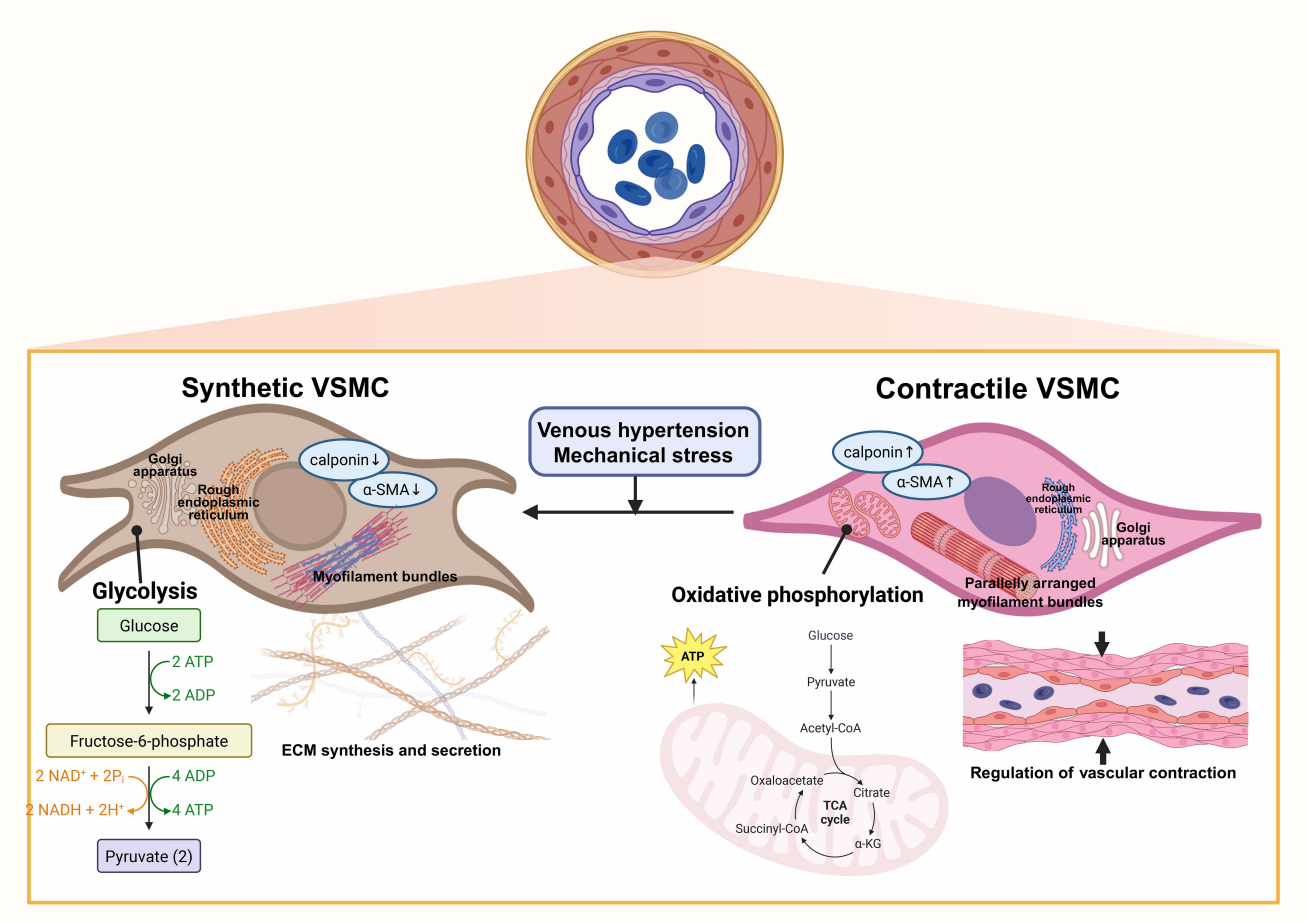
Characteristics and metabolic profiles of synthetic and contractile VSMCs. Comparative illustration of the phenotypic and metabolic shifts in VSMCs during varicocele pathogenesis. The left panel depicts synthetic VSMCs, characterized by developed organelles for secretion, reliance on glycolysis, and expression of synthetic markers. The right panel shows contractile VSMCs, featuring abundant myofilaments, reliance on oxidative phosphorylation for efficient energy production, and expression of contractile proteins. The transition from a contractile to a synthetic phenotype, driven by venous hypertension and mechanical stress, is a key event in venous wall remodeling and loss of tone.

Abnormal signals from the intima (inflammatory cytokines, oxidative stress products) can act on the media, inducing metabolic disturbances characterized by VSMC phenotypic switching (Cao et al., 2022). As the primary cellular source of collagen and elastic fibers in the vascular wall, the phenotypic switching of VSMCs plays a central role in varicose vein pathogenesis. Under pathological conditions, VSMCs transition from a quiescent contractile phenotype to an activated synthetic phenotype. This shift is characterized by downregulation of contractile markers (e.g., α-SMA, SM22α) and upregulation of synthetic markers (e.g., OPN, vimentin), leading to excessive ECM synthesis (Ghaderian and Khodaii, 2012; Li et al., 2026). This phenotypic conversion yields two critical consequences: disruption of collagen balance and loss of contractile function in VSMCs, impairing the venous wall’s capacity for tension regulation (Jacob, 2003; Sansilvestri-Morel et al., 2003). Advanced venous wall remodeling may involve irreversible VSMC loss and ECM disruption, which might indicate that early intervention and therapies targeting VSMC phenotypic switching to promote contractile over synthetic phenotypes are desirable. Characteristics of synthetic and contractile VSMCs are seen in **Table 1** and **Figure 3**.

**Table 1:** Characteristics of synthetic vs. contractile VSMCs in vascular pathology.

Feature	Contractile VSMCs (Sobue et al., 1999; Rzucidlo et al., 2007)	Synthetic VSMCs (Campbell and Campbell, 1985; Owens et al., 2004; Werle et al., 2005; Allahverdian et al., 2018)
Proliferative Capacity	Low	High
Migration Capacity	Low	High
Primary Function	Vascular contraction, regulation of vascular tone	Synthesis of ECM, involvement in repair and pathological remodeling
Metabolic Characteristics	Dependent on oxidative phosphorylation (OXPHOS), fatty acid oxidation (FAO)	Aerobic glycolysis (Warburg effect), enhanced pentose phosphate pathway (PPP)
Energy Production	High efficiency (36 ATP/glucose)	Low efficiency (2 ATP/glucose), but provides biosynthetic precursors
Typical Environmental Context	Healthy vascular media	Pathological conditions such as vascular injury, atherosclerosis, hypertension, aneurysms

Synthetic VSMCs produce abundant protease inhibitors, slowing degradation of newly formed collagen fibrils (Jacob, 2003). However, as vascular wall stiffening intensifies, VSMC numbers decline, and the muscular layer undergoes progressive replacement by collagen fiber bundles. This results in structural disruption of the tunica media, establishing a self-depleting vicious cycle that ultimately abolishes the functional integrity of the muscular layer (Studennikova et al., 2019). Ultrastructural evidence supports this progression. In a study of 25 male patients (mean age 19 years), biopsy samples from varicose spermatic veins and lower extremity veins showed analogous changes: collagen fiber bundle disarray, reduced VSMC volume, and connective tissue hyperplasia, indicating functional incompetence of the connective tissue framework in VC pathogenesis (Studennikova et al., 2020). Additional morphological alterations in the pampiniform plexus of VC patients include reduced longitudinal and oblique SMCs, increased connective tissue components, and diminished circular SMCs in the media (Macchi et al., 2008). These changes are considered the structural basis for loss of venous wall integrity and tone.

Notably, different VC grades exhibit progressive pathological stages. Grade I shows no significant morphological abnormalities; grade II is characterized by venous wall thickening (hyperplasia); and grade III presents with marked luminal dilation (Yildiz et al., 2003). Wall thickness in both grades II and III is significantly greater than in grade I. Mechanistically, endothelial NO dysregulation abolishes its inhibitory effect on smooth muscle proliferation. Combined with sustained hypertensive stretch, this leads to compensatory upregulation of α1−adrenergic receptors, enhanced contractile responsiveness, and smooth muscle hyperplasia, resulting in wall thickening (grade II). When persistent hypertension exceeds compensatory capacity, smooth muscle collagen and elastic fibers fracture, rendering contraction ineffective and leading to loss of wall support and luminal dilation (grade III). Moreover, inflammatory and vasoconstrictive mediators released by decompensated smooth muscle cells further damage the endothelium, exacerbating smooth muscle dysfunction and establishing a vicious cycle (Yildiz et al., 2003). This may also explain the increased expression of contractile phenotype markers in grade III VC observed by Wenxin Li et al., potentially reflecting maladaptive fibrosis and hypercontractility rather than functional recovery (Li et al., 2026).

## 6 Adventitial pathology: driver of chronic inflammation and pathological fibrosis

The adventitia, the outermost layer of the venous wall, consists of loose connective tissue, fibroblasts, collagen fibers, elastic fibers, vasa vasorum, and nerve endings. Under physiological conditions, the adventitia provides structural support and maintains vascular compliance (Gibb et al., 2020). In the pathological microenvironment of VC, we propose that the adventitia serves as the primary anatomical site for chronic inflammation and progressive fibrosis, driven by sustained signals from the dysfunctional intima and media.

While direct histological studies of VC adventitia remain limited, evidence from related venous hypertension models—specifically great saphenous vein varicosities—demonstrates that the adventitia harbors activated progenitor cells (CD34+CD117+) involved in vascular remodeling and inflammatory cascades (Ling et al., 2021). In the context of VC, ROS and inflammatory cytokine levels within the spermatic vein are reported to be significantly elevated in VC and correlate positively with the degree of venous dilation (Yoon et al., 2010; Lattimer et al., 2016). Given that ROS are key drivers of both inflammation and fibroblast activation, these findings suggest a pro-inflammatory and pro-fibrotic milieu within the VC adventitia, though direct validation is required.

Resident adventitial fibroblasts become activated under the combined stimuli of inflammatory cytokines and mechanical stress, such as increased venous wall tension. They undergo phenotypic conversion into myofibroblasts, cells that possess both the synthetic capacity of fibroblasts and the contractile properties of smooth muscle cells and serve as the central effectors of fibrosis (Stenmark et al., 2013). However, direct confirmation of this phenotypic transition within the varicocele venous wall is currently lacking and warrants further investigation. Activated myofibroblasts synthesize and secrete excessive amounts of ECM components, predominantly collagen types I and III (Sansilvestri-Morel et al., 2003). These collagen fibers are often deposited in a disorganized manner, leading to progressive adventitial fibrosis. Although this fibrotic process thickens the vessel wall, it simultaneously abolishes elasticity, reduces compliance, and renders the wall stiff, thereby further compromising venous function. In the spermatic veins of patients with VC, histopathological observations confirm the presence of a thickened connective tissue layer in the adventitia, a reduction in oblique muscle fibers, and fibrous degeneration (Muxitdinovich and Daminovich, 2019). Rather than strengthening the vessel, this adventitial fibrosis paradoxically results in loss of compliance and increased venous stiffness, further compromising venous function. It is plausible to speculate that the thickened and fibrotic adventitia mechanically compresses the vasa vasorum and nerve plexuses within it, thereby impairing venous wall perfusion and stimulating adjacent nerve endings. If validated, this mechanism could account for the scrotal pain frequently observed in VC, with nociceptive signals presumably arising from entrapped adventitial nerves (Lai et al., 2023).

**Figure 4:**
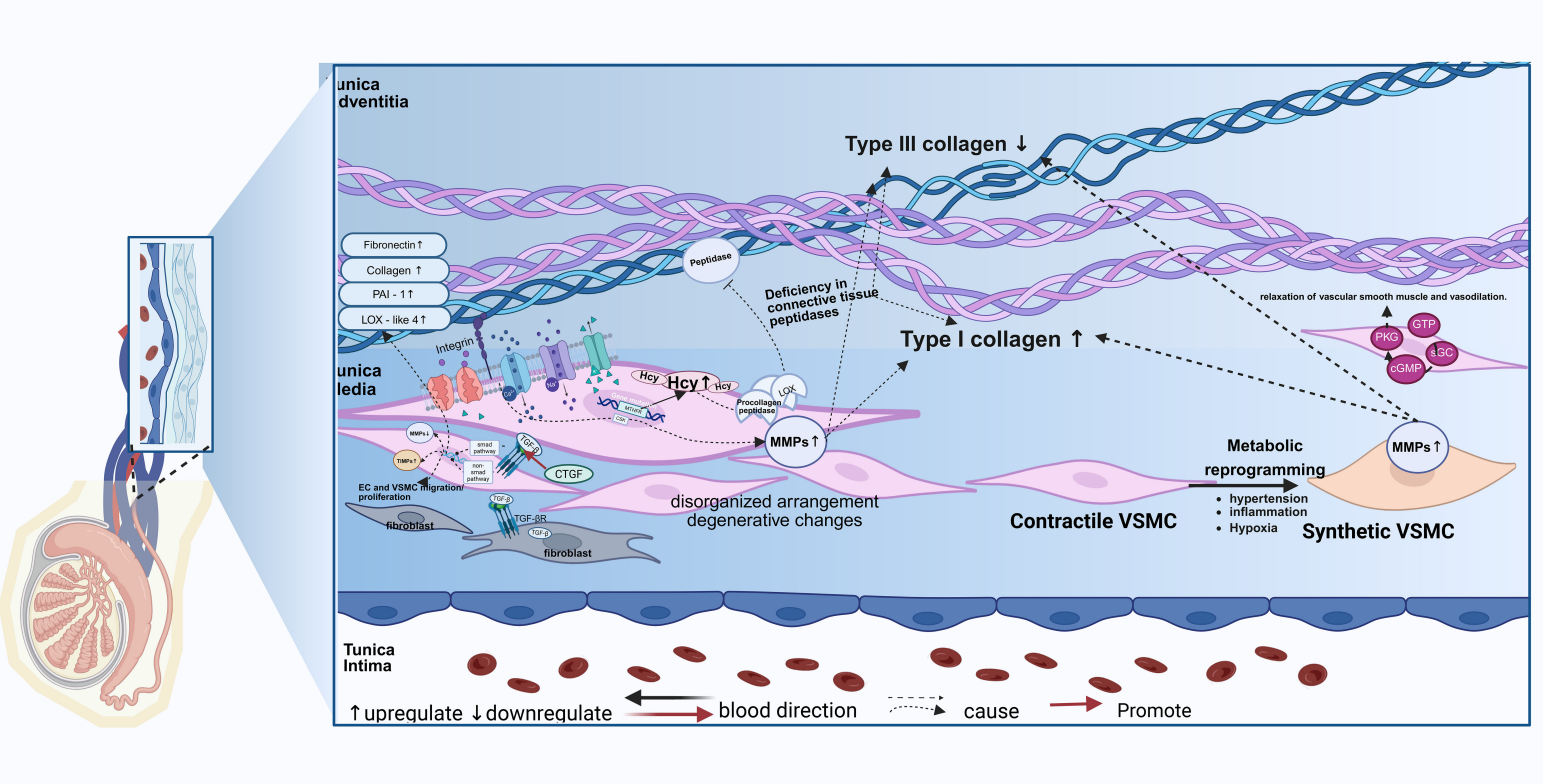
Main potential mechanisms of venous wall remodeling in varicocele. Venous wall remodeling in varicocele primarily involves ECM dysregulation and VSMC dysfunction. Genetic factors (e.g., FOXC2, MTHFR) and venous hypertension activate HIF-1α/2α, upregulating MMP-2/9. This disrupts collagen balance (↑Type I, ↓Type III), reduces elastin, and degrades ECM integrity. MMPs further impair venous tone by inducing VSMC relaxation and endothelial damage. Paradoxically, TGF-β1 expression is reduced, suggesting compensatory inhibition of its profibrotic role. VSMCs exhibit phenotypic switching from contractile to synthetic states, losing α-SMA expression and contractile function. This promotes aberrant ECM production and reduces venous wall tension. Advanced stages feature VSMC depletion and medial layer collagen replacement, perpetuating venous dilation.

Adventitial remodeling of the vessel wall may have a genetic predisposition. The FOXC2 gene was the first identified to be associated with insufficiency in both superficial and deep venous valves (Ng et al., 2005). The C677T functional polymorphism of the MTHFR gene is associated with varicose vein development, with carriers at increased risk of disease. This gene regulates homocysteine levels, thereby impacting vascular wall integrity (Karathanos et al., 2013). Genetically determined connective tissue peptidase deficiency impairs type III collagen synthesis, leading to reduced type III collagen and increased type I collagen. This compromises structural support, serves as the basis for venous wall and valve collapse, and induces further alterations in extracellular matrix components, inevitably exacerbating venous insufficiency (Sansilvestri-Morel et al., 2003) However, the expression of related genes and enzymes in varicocele remains scarce. There is also an alternative perspective. The molecular underpinnings of this adventitial fibrosis, while not yet fully characterized in VC specifically, likely involve dysregulated ECM turnover driven by venous hypertension (Atta, 2012). Prolonged mechanical stress stimulation induces HIF-1α/HIF-2α overexpression and elevates MMP-2/MMP-9 levels (Raffetto et al., 2008), subsequently triggering increased type I collagen deposition and degradation of type III collagen (Lim and Davies, 2009). **Figure 4** provides a schematic overview of the principal mechanisms underlying this venous wall remodeling. This remodeling signature, extensively documented in chronic venous insufficiency (CVI), results in progressive loss of venous wall compliance and irreversible dilation. While VC shares fundamental hemodynamic features with CVI, namely venous valve dysfunction and localized hypertension, direct evidence confirming this specific MMP-mediated collagen shift in the VC venous wall remains absent. Although MMP-9 has been identified as a common molecular factor in VC, inguinal hernia, and lower extremity varicosities (Serra et al., 2014), its precise contribution to VC adventitial fibrosis and symptom development remains to be investigated.

In certain studies, Transforming Growth Factor Beta 1 (TGF-β1) is implicated as a TIMP stimulator in CVI (Ruiz-Ortega et al., 2007; Serralheiro et al., 2017). Under pathological conditions, they inhibit MMP-mediated degradation of the ECM, thereby reducing elastin content and increasing venous wall stiffness (Serralheiro et al., 2017). Concurrently, they hyperactivate VSMCs and fibroblasts via pathways such as TGF-β1/Smad3, promoting excessive synthesis and deposition of collagen and fibronectin (Ryer et al., 2006).

Similarly, the TGF-β1/Smad signaling axis, a potent driver of fibrosis and TIMP-mediated ECM stabilization in other venous beds, presents a conflicting and incompletely characterized picture in VC. While elevated TGF-β1 is detectable in the serum and testicular parenchyma of VC patients, its expression and functional activity within the VC venous wall adventitia have not been quantified (Dobashi et al., 2002). Therefore, while pharmacological inhibition of MMPs or TGF-β1 signaling represents a compelling theoretical strategy to halt venous wall fibrosis, these concepts currently constitute hypothesis-generating avenues rather than clinically validated targets for VC intervention.

Some studies emphasize the active role of the adventitia in driving whole−vessel wall remodeling through an outside−in signaling process. Activated adventitial fibroblasts or myofibroblasts in damaged vessels release large amounts of cytokines (e.g., IL−6), growth factors, and ROS, which can diffuse inward and affect medial smooth muscle cells and intimal endothelial cells (Dutzmann et al., 2025). However, the endothelial injury described in those studies is mostly induced by artificial mechanical arterial damage (e.g., following percutaneous coronary intervention), which differs considerably from the pathological environment of VC. Therefore, we still favor the inside−out signaling and conduction model as the driver of venous wall remodeling in VC. However, the precise situation requires direct validation in VC models.

## 7 From vascular wall pathology to testicular injury

Within the theoretical framework advanced herein, the sustained progression of venous wall pathology culminates in two irreversible structural consequences. First, the venous valve, an extension of the intima, depends critically upon medial mechanical support for proper leaflet coaptation. As fibrotic remodeling of the vessel wall intensifies, the valve leaflets undergo progressive thickening, retraction, and eventual organization, thereby transforming valvular incompetence from a reversible functional impairment into a fixed structural lesion that perpetuates reflux irrespective of transient fluctuations in intraluminal pressure (Studennikova et al., 2019). Second, the venous wall, deprived of elastic recoil and encumbered by disorganized ECM deposition, can no longer withstand persistent intraluminal pressure, resulting in permanent dilation and tortuosity that manifests clinically as the visible “bag of worms” configuration of the pampiniform plexus (Macchi et al., 2008).

Viewed through this lens of structural venous wall failure, the classic pathways leading to testicular injury can be logically reintegrated and afforded deeper mechanistic coherence. Venous stasis and reflux are accordingly reframed not as isolated hemodynamic events but rather as the direct consequence of weakened intimal-medial support at the valve attachment sites (Alperen et al., 2022). The phenomenon of scrotal hyperthermia, long recognized as a central pathogenic factor, is hypothesized to stem largely from the impaired contractile function of smooth muscle within the remodeled tunica media of the pampiniform plexus, which compromises the countercurrent heat exchange essential for testicular thermoregulation (Ismail et al., 2014). Similarly, the persistence of oxidative stress and the retrograde flux of toxic metabolites are traceable to the diseased vessel wall itself: activated endothelial cells, infiltrating inflammatory leukocytes, and pro-fibrotic adventitial fibroblasts collectively serve as potent sources of ROS and inflammatory mediators, while the concomitant disruption of endothelial barrier integrity facilitates the pathologic reflux of adrenal- and renal-derived metabolites into the testicular microenvironment (Schoor et al., 2001; Wang et al., 2025). Germ cell apoptosis, Sertoli cell dysfunction, and impaired Leydig cell steroidogenesis thus represent the terminal manifestations of this cumulative, multi-hit cascade (Ammar et al., 2021). This reconstructed pathogenetic framework suggests that VC is more appropriately understood as a primary venopathy of the spermatic cord, wherein secondary testicular dysfunction arises from the sustained biological activity and mechanical incompetence of a structurally compromised venous wall.

## 8 Conclusion

This review elucidates the pathophysiological mechanisms of VC from a venous vasculature perspective. VC pathogenesis involves remodeling of the venous wall across three layers, as shown in **Table 2**. Adventitia is characterized by dysregulated ECM metabolism, including collagen imbalance, elastin reduction, and aberrant MMP/TIMP activity. Media centers on VSMC phenotypic switching from contractile to synthetic phenotype, leading to loss of wall tension and progressive dilation. Intima features endothelial dysfunction driven by glycocalyx impairment, oxidative stress, HIF-1α/VEGF activation, and inflammatory pathways. It shifts the research focus from testicular damage to the spermatic vein itself, establishing venous structural and functional impairment as the central pathophysiological mechanism in VC. It moves beyond isolated, mechanistic descriptions by integrating hemodynamic disturbances, ECM remodeling, oxidative stress, chronic inflammation, and neuroendocrine signaling into a coherent pathological network. This might suggest that future research could extend beyond simply blocking reflux to include assessment and intervention targeting the venous wall. For example, new imaging techniques could be explored to evaluate the biomechanical properties of the venous wall, and drugs that modulate endothelial function or affect pathological remodeling processes could be investigated as potential adjunctive therapies. Incorporating both macroscopic hemodynamic factors and microscopic venous wall changes into a comprehensive framework could perhaps offer new insights for the diagnosis and treatment of VC.

**Table 2:** Representative pathological mechanisms and potential therapeutic targets in VC.

Vessel layer	Mechanism Overview	Main Pathways	Research evidence description	Potential therapeutic strategy	Pending research questions	Proposed experimental strategy
Intimal Injury	Endothelial dysfunction (Gyftopoulos et al., 2011; Kiziler et al., 2015; Boulanger, 2016; Wang et al., 2021; Zhang et al., 2021)	Glycocalyx damage increases endothelial permeability	Inferred from CVD studies	Sulodexide(glycosaminoglycan complex)	The effects of Sulodexide on glycocalyx repair, MMP-9 inhibition, and reduction of inflammatory infiltration in VC need verification.	*In vitro*: TEM/lectin staining of HSVECs ± Sulodexide. *In vivo*: IHC for CD45/MMP-9 in rat VC model.
		NO metabolic imbalance	Serum detection study of spermatic vein; Partial mechanisms supported by related animal model studies; Complete mechanism inferred from general theories of vascular diseases	BH4 stabilizers (Folate/antioxidants)	The existence of eNOS uncoupling in VC models needs confirmation, and whether BH4 stabilizers can restore NO production and reduce ROS requires verification.	Ex vivo: Low-temperature SDS-PAGE for eNOS dimer/monomer ratio. *In vivo*: DHE staining for ROS after Sepiapterin treatment.
		ET-1 and ETA/ETB receptor upregulation	Direct study of spermatic vein	Receptor antagonists (e.g., Bosentan)	The specific role of the ET-1/receptor axis in VC venous remodeling needs clarification, and the therapeutic potential of receptor antagonists requires assessment.	Human tissue: Laser-capture microdissection + qPCR for ETA/ETB. *In vivo*: Bosentan treatment in rat VC model.
		HIF-1α/VEGF signaling axis activation	Animal model study of spermatic vein	HIF inhibitors	Its role in VC remains unclear; exploration is needed to determine if it acts by blocking hypoxia-induced MMP upregulation and vascular remodeling.	Genetic: Smooth muscle-specific Hif1a KO mice. Readout: *In situ* zymography for MMP activity.
		Elevation of pro-inflammatory factors such as IL-1β, IL-6, and TNF-α, and activation of the NF-κB/MAPK pathways	Integrated with spermatic vein blood and seminal plasma data, inferences based on general inflammation theory.	Anti-TNF agents (e.g., Infliximab)/IL-1 receptor antagonists	The efficacy of targeting specific cytokines in VC needs evaluation, and whether it acts by blocking the NF-κB/MAPK pathway requires clarification.	*In vivo*: Etanercept in VC rats. Readout: Western blot for p-NF-κB p65 in venous tissue.
Medial Remodeling	VSMCs dysfunction (Jacob, 2003; Studennikova et al., 2020; Cao et al., 2022)	VSMC disorganized distribution, degeneration, and phenotypic switch from contractile to synthetic	Observational study of spermatic vein; Mechanisms inferred from research on lower limb varicose veins and other venous disorders	Promote contractile phenotype	Direct evidence of VSMC phenotypic switching in VC is lacking; its initiating mechanisms and contribution to loss of venous wall tension need investigation.	Human tissue: scRNA-seq of pampiniform plexus cells. Mouse: Lineage tracing (*Myh11-CreERT2; tdTomato*).
	ECM remodeling (Sansilvestri-Morel et al., 2003; Lim and Davies, 2009; Serra et al., 2014)	Collagen imbalance;elastin degradation, high expression of MMP-2/9; TIMP imbalance	High MMP-9 expression, from direct study of spermatic vein; Others inferred from theories of VVLE and CVI, etc.	TIMP enhancers, Synthetic inhibitors (Batimastat/Marimastat), Doxycycline	Efficacy and specific mechanisms need validation in VC models, e.g., whether it alleviates venous dilation by inhibiting collagen/elastin degradation.	*In vivo*: Doxycycline in rat VC model. Readout: Desmosine assay for elastin degradation.
Adventitial Fibrosis	Collagen Disorder (Lim and Davies, 2009; Stenmark et al., 2013; Muxitdinovich and Daminovich, 2019)	Abnormality in TGF-β1/Smad3 pathway, regulates ECM synthesis and fibrosis	TGF-β1 changes in VC serum/testis tissue; pathway protein Expression/regulation inferred from CVI studies	TGF-β receptor kinase inhibitors, Anti-CTGF mAb, STAT3 inhibitors	The expression changes and net effect of the TGF-β1 signaling pathway in VC are controversial; its specific role and regulatory mechanisms need clarification.	Human tissue: Phospho-Smad2/3 IHC. *In vivo*: SB-431542 treatment + Masson’s trichrome.

However, this study has several limitations. Many described cellular and molecular mechanisms are primarily extrapolated from models of lower extremity venous insufficiency or arterial diseases. Although we have carefully considered their relevance and noted differences, this may still affect the accuracy of our mechanistic interpretations. The spermatic veins constitute a unique vascular bed, and tissue-specific cellular responses in VC remain unverified. Furthermore, current animal models inadequately recapitulate the chronic progression, distinct hemodynamics, and subsequent venous wall remodeling seen in humans. The involvement of neuroendocrine factors primarily relies on preliminary clinical data, with their cellular origins and signaling networks remaining unclear.

Additionally, while VC exhibits clinical heterogeneity, our review does not fully address how the proposed mechanisms vary across clinical subtypes. Proposed therapeutic strategies lack validation in varicocele-specific studies, and the risk-benefit profile of repurposing potent biologics for this benign condition requires careful evaluation. Finally, some cited references are dated but were included for their foundational relevance.

Future research should focus on the following directions: (1) validating key mechanisms by defining ECM metabolic networks, clarifying the role of TGF-β1 signaling, directly demonstrating VSMC phenotypic switching, and confirming glycocalyx impairment, NO-ROS imbalance, HIF-VEGF axis dysregulation, and inflammasome activation in the spermatic vein to construct a comprehensive pathological network. (2) Actively exploring novel, precise therapeutic strategies targeting these pathways. This includes testing the efficacy of MMP inhibitors, specific antioxidants, and anti-inflammatory agents in preclinical models to assess their potential to improve venous function and fertility outcomes. (3) Recognizing the progressive nature of the disease, it is essential to correlate pathophysiological changes with clinical grading and focus on the dynamic alterations that occur during disease progression. (4) Identifying specific biomarkers (e.g., specific miRNAs, nitrated proteins, and MMP levels) in spermatic venous blood or peripheral blood that can accurately reflect the degree of venous wall injury and oxidative stress, thereby aiding in the precise diagnosis and assessment of VC. (5) Exploring advanced therapeutic platforms such as nanoparticle-based targeted delivery and RNA-based therapeutics. (6) Identifying biomarkers of venous wall exhaustion may enable a biology-based staging system to complement clinical grading. (7) Gene editing and cell therapy strategies include reprogramming induced pluripotent stem cells (iPSCs) via transcription factors to generate organ-specific endothelial cells for endothelial repair; utilizing mesenchymal stem cells (MSCs) to transfer mitochondria to injured endothelium, restoring ATP production and metabolic homeostasis, among others (Augustin and Koh, 2024). These platforms have demonstrated significant potential in other vascular disease areas but represent a virtually untapped field of research for VC application.
